# Bit-related lesions in Icelandic competition horses

**DOI:** 10.1186/s13028-014-0040-8

**Published:** 2014-08-13

**Authors:** Sigríður Björnsdóttir, Rebecka Frey, Thorvaldur Kristjansson, Torbjörn Lundström

**Affiliations:** 1 Icelandic Food and Veterinary Authority, Austurvegur 64, Selfoss, IS-800, Iceland; 2 Norsholms Djursjukhus, Biskop Henriksv 6, Norsholm, S-610 21, Sweden; 3 Agricultural University of Iceland, Hvanneyri, Borgarnes, IS-311, Iceland; 4 Djurtandvårdskliniken, Västra Husby, Norrköping, S-605 96, Sweden

**Keywords:** Oral lesions, Ulcer, Bit, Carriage, Oral examination, Equine, Animal welfare

## Abstract

**Background:**

Oral lesions related to the use of the bit and bridle are reported to be common findings in horses worldwide and represent an important animal welfare issue. In order to provide an overview of bit-related lesions in Icelandic competition horses, a field examination of the rostral part of the oral cavity was performed in 424 competition horses coming to the two major national horse events in Iceland in 2012. Records from repeated examination of 77 horses prior to the finals were used to assess potential risk factors.

**Results:**

Mild lesions were recorded in 152 horses (36%) prior to the preliminary rounds. They were most often located in the commissures of the lips and the adjacent buccal mucosa (n = 111). Severe lesions were found in 32 (8%) horses. For 77 horses examined prior to the finals, the frequency of findings in the area of the mandibular interdental space (bars of the mandible) had increased from 8% to 31% (*P* < 0.0001). These findings were most often (16/24) regarded as severe. The presence of lesions on the bars was strongly associated to the use of curb bits with a port (OR = 75, *P* = 0.009).

**Conclusions:**

Bit-related lesions were found to be a general problem in Icelandic competition horses. The type of bits used influenced both the location and the severity of the lesions. The use of curb bits with a port was found to be a decisive risk factor for lesions on the bars of the mandible, most of which were regarded as severe. The results also raised questions about the head and neck carriage demanded for the competition horses.

## Background

Ulcers and abrasions of the buccal mucosa, the lips and the tongue are common findings in horses worldwide [1]-[5]. Ulcers located adjacent to the first and second premolar teeth and in close proximity to the commissures of the lips, are reported to be caused by the stress of bit and bridle, without being influenced by routine floating of the teeth [5]. Bit-induced lesions located on the bars of the mandible are also described, including soft tissue oedema, erosions to the mucosa, periosteal reactions and exostoses, and regarded to be of concern for animal welfare [6],[7].

The Icelandic horse is used for pleasure riding and gait competitions. In addition to the naturally occurring gaits in all equids - walk, trot and canter/gallop - the performance of the lateral four-beat gait *tölt*, is characteristic for the breed. Many Icelandic horses also have the ability to pace. The characteristics of the different gaits and the special gait competitions developed for the Icelandic horse have been thoroughly described by Albertsdottir *et al*. [8] and detailed information about the judging rules is available online [9].

Separate disciplines exist for five-gaited horses (performing walk, trot, canter, *tölt* and pace) and four-gaited horses (performing walk, trot, canter and *tölt*). A four gaited discipline is also offered for young-riders (in the age range of 17–21). Other disciplines include the special *tölt* competitions where T1 is the test with the highest demand regarding collection, head and neck carriage and limb action at the most extreme speed ranges (performing slow *tölt*, speed changes and fast *tölt*). In T2, the horses perform slow *tölt* and free speed *tölt*, but must in addition be able to perform *tölt* at slow to medium speed without rein contact. A special pace test (judged on both speed and gait quality) and pace races are also among commonly practiced disciplines.

The aim of the study was to provide an overview of bit-related lesions in Icelandic competition horses and to assess potential risk factors that can be used for prevention of the lesions.

## Methods

### Horses

Examination of the rostral part of the oral cavity was compulsory according to the rules of the Icelandic Equestrian Association and performed according to the same protocol for all competition horses participating in the two major national horse events in Iceland in 2012. The horses were examined prior to the first preliminary rounds of competition and those who qualified for the finals were subject to a repeated examination prior to the finals. This provided data from 334 horses competing at the National horse event (*Landsmót hestamanna 2012*) and 90 horses competing at the National event for equestrian sport (*Íslandsmót í hestaíþróttum 2012*), in total 424 horses, whereof 77 had two records (Table 1). For horses that competed in more than one discipline in the preliminary rounds, only the results from the first examination were used for data analysis. Usually, each horse qualified for the finals in only one discipline. For horses that qualified for the finals in the same discipline in the two major national horse events, the results from the first examination (prior to the first preliminary round) and the last one (prior to the later final round) were used and the number of performances in the meantime (including participation in other disciplines and shows) was recorded.

**Table 1:** Overview of bit-related lesions in Icelandic competition horses examined prior to the preliminary rounds

		**Bit-related lesions**				
		**Mild**		**Severe**		**Total (%)**
**Discipline**	**n**	**Buccal**	**Bars**	**Buccal**	**Bars**	
5-gaited tests	125	39	16	3	3	61 (49)
4-gaited tests	117	37	6	6	5	54 (46)
Young-riders	81	22	3	6	2	33 (41)
T1	27	3	4	3	3	13 (48)
T2	8	0	1	0	0	1 (13)
Pace test/races	66	10	11	0	1	22 (33)
Total (%)	424	111 (26)	41 (10)	18 (4)	14 (3)	184 (43)

The variation in the frequency of severe lesions deviated significantly between different disciplines in the preliminary rounds, tested by χ^2^ tests with four degrees of freedom (df) (*P* = 0.0104).

### Oral examination

**Figure 1:**
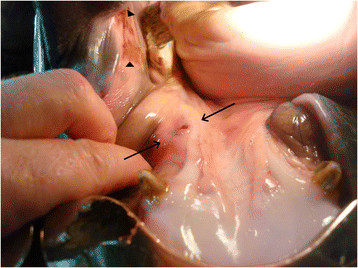
**Mucosal inflammation and ulceration of the bar of the mandible combined with pressure lesions of the buccal mucosa.** Arrows: Inflammation and ulcer in the mucosa of the bar. Arrowheads: Pressure lesions on the buccal mucosa.

**Figure 2:**
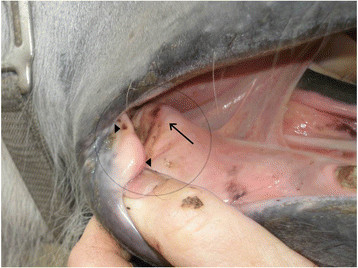
**A pressure lesion overlies a prominent thickening of the bar of the mandible, the thickening indicating severe periostitis.** Adjacent is a thickening of the buccal mucosa with a pressure lesion in close proximity to the corner of the mouth. Arrow: Prominent thickening of the bar with a pressure lesion. Arrowheads: Thickening of the mucosa with lesions.

The horses were examined outdoors at the competition area, 1–24 hours before competing. A team of eight veterinarians with experience in oral examination of horses performed the examinations, coordinated by the veterinarian in charge. It consisted of inspection and palpation of the oral mucosa and underlying tissues of the rostral part of the mouth. Taking the tongue to the outside of the mouth facilitated the procedure – neither an oral speculum nor sedation was used. When the tongue was taken to one side of the mouth it allowed examination of the contralateral side and vice versa. The location of the lesions was recorded and then classified as occurring in the buccal region (the commissures of the lips or the buccal mucosa), in the bar region (the mucosa covering the mandibular interdental space, the underlying connective tissue and bone) or on the tongue. The lesions were graded as mild (thickening of the mucosa, superficial pressure lesions or small ulcers, up to 1 cm in diameter, without detectable soreness or inflammation) or severe (ulcers in the mucosa more extensive than 1 cm in diameter, inflammation and/or soreness of the mucosa, prominent thickening of the bars), (Figures 1 and 2). In the case of lesions in more than one location, the most severe one counted in the classification. If equal in grading, lesions located at the bar region counted in the classification.

### Potential risk factors

**Figure 3:**
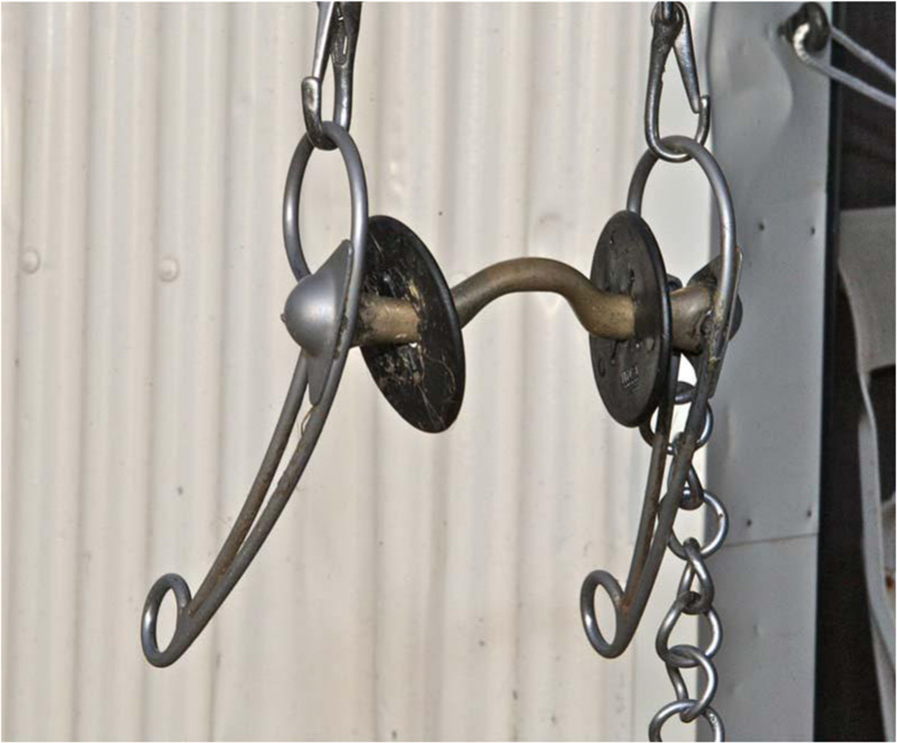
Curb bit with a port, unjointed.

**Figure 4:**
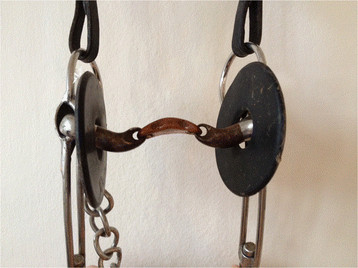
Curb bit with a port, double jointed.

**Figure 5:**
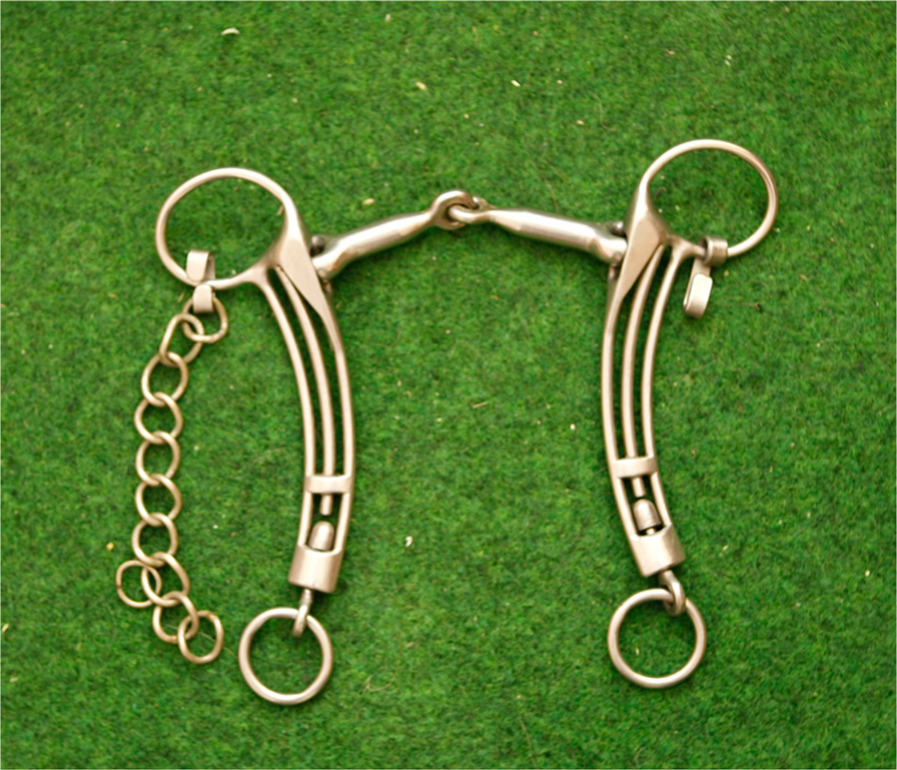
Traditional Icelandic curb bit without a port single jointed.

For horses that qualified for the finals, information about the bits and nosebands used in the performance prior to the second examination (1–5 days earlier) was collected by interviewing the riders (n = 25), video tapes (n = 44) or other applicable footage (n = 8). The most commonly used bits, 45 out of 77, (58%) were curb bits with a port (unjointed or jointed) (Figures 3 and 4) in combination with an English noseband in all cases except two, where no noseband was used. Twenty-six horses (34%) were ridden with different types of snaffle bits (the particular types of snaffle bits were not identified) in combination with German/Hanoverian or double nosebands. Lever nosebands were used in two cases. Six horses (8%) were ridden with traditional Icelandic curb bits (Figure 5) and always combined with English nosebands. Use of the English noseband was common for the two types of curb bits and use of the German noseband was restricted to snaffle bits, therefore, only the type of bit could be assessed as a risk factor.

The 77 horses that qualified for the finals were ridden by 54 different riders (38 riders rode one horse, 10 riders rode two horses, five riders rode three horses and one rider rode four horses). The effect of the rider was not included in the statistical model due to few riders who rode multiple horses and because of the confounding effect between the bit and rider.

### Statistical analysis

Statistical analyses were performed using the statistical package SAS (version 9.2) [10]. To investigate whether the frequency of oral lesions (mild, severe and total) deviated significantly between different disciplines in the preliminary rounds, χ^2^ tests with four degrees of freedom (df) were performed with the Proc Freq procedure [10].

For assessment of potential risk factors binominal outcome variables (lesions vs no lesions) were used. Generalized estimating equation (GEE), developed by Liang & Zeger, was applied [11] for analysis of repeated measurements of 77 horses, where the variable ‘examination’ was the only fixed factor. Separate analyses were performed for lesions in the buccal and bar regions. The Proc Genmod procedure in the statistical package SAS (version 6.12) [10] was used. The link-function in Proc Genmod was specified as logistic as binominal outcome variables for lesions in the buccal and the bar regions of the mouth were used. Multivariable logistic regression was used to estimate the association between disciplines, bits and number of performances and the binomial dependent variables. Two-way interactions between descriptive variables were tested. For the logistic regressions the Proc Logistic procedure was used [10].

## Results

### Examination of 424 horses prior to the preliminary events

Mild oral lesions were found in 152 horses (36%) prior to the preliminary tests, most often located on the inside of the commissures of the lips and in the adjacent buccal mucosa (n = 111). The mild findings in the buccal region were characterized by hyperkeratosis surrounding an ulcerated centre, indicating a chronic history. Mild findings in the bar region (n = 41) included mild thickening of the mucosa and the underlying connective tissue and superficial pressure lesions in the mucosa.

Thirty-two horses (8%) had severe lesions at the first examination - 18 horses had ulcers in the buccal region and 14 horses had findings in the bar region. The severe lesions in the bar region ranged from prominent thickening of the mucosa and the underlying connective tissue, sometimes with oedema, to ulcerations in the mucosa, periostitis and remodelling of the bone.

The frequency of severe lesions was found to be lower in horses competing in T2 and pace tests compared to the other disciplines (*P* = 0.0104) (Table 1).

### Repeated examination of 77 horses

Re-examination performed prior to the finals revealed bit-related lesions in 60% of the horses. The frequency of lesions in the bar region increased between the two examinations from 8% to 31% (OR = 9.25, *P* = 0.0002) while no change was found for lesions in the buccal region (Tables 2 and 3). Lesions in the bar region were found in 51% of horses ridden with a curb bit which had a port but only in one horse (4%) ridden with a snaffle bit. On the other hand, 62% of horses ridden with a snaffle bit had lesions in the buccal region compared to 13% of horses ridden with curb bits which had a port. Six horses ridden with traditional Icelandic curb bits were without findings in both locations. Sixteen out of 24 findings recorded in the bar region at the second examination (67%) were regarded as severe compared to 2 out of 22 findings in the buccal region (9%). The number of performances between the two examinations varied from one to six with a mean of 2.2.

**Table 2:** Multivariable analysis of potential risk factors for lesions in the buccal region of 77 Icelandic competition horses that qualified for the finals

**Risk factor**	**n**	**Frequency (%)**	***P*-value**	**OR**^**c**^	**95% CI**^**d**^
**Examination**^**a**^			0.4965		
First examination	77	33			
Second examination	77	29			
**Discipline**^**b**^			0.9734		
T1	12	17			
T2	5	0			
Young riders	15	40			
Four-gaited	23	35			
Five-gaited	22	27			
**Bit**^**b**^			0.0002		
Snaffle bit	26	62		8.07	2.38 - 27.44
Icelandic curb bit	6	0		-	-
Curb bit with a port	45	13		1.00	
**Number of performances**^**b**^			0.8907		
1	21	33			
2	34	21			
3	14	36			
≥4	8	2			

^a^Results regarding examination are from the GEE analysis.

^b^Results regarding test, bridle and number of performances are from the multivariable logistic regression analysis.

^c^OR refers to odds ratios.

^d^CI refers to confidence interval.

**Table 3:** Multivariable analysis of potential risk factors for lesions in the bar region of 77 Icelandic competition horses that qualified for the finals

**Risk factor**	**n**	**Frequency (%)**	***P*-value**	**OR**^**c**^	**95% CI**^**d**^
**Examination**^**a**^			<0.0001		
First examination	77	8		1.00	
Second examination	77	31		9.29	3.03 - 28.50
**Discipline**^**b**^			0.413		
T1	12	67			
T2	5	0			
Young riders	15	20			
Four-gaited	23	26			
Five-gaited	22	32			
**Bit**^**b**^			0.009		
Snaffle bit	26	4		1.00	
Icelandic curb bit	6	0		-	-
Curb bit with a port	45	51		75.1	4.29 - 867.25
**Number of performances**^**b**^			0.239		
1	21	14			
2	34	32			
3	14	43			
≥4	8	50			

^a^Results regarding examination are from the GEE analysis.

^b^Results regarding test, bridle and number of performances are from the multivariable logistic regression analysis.

^c^OR refers to odds ratios.

^d^CI refers to confidence interval.

The multivariable analysis revealed a strong association between the type of bits and the risk of oral lesions. Horses ridden with curb bits which had a port had 75 times higher odds for lesions on the bars (*P* = 0.009) (Table 3) compared to horses ridden with snaffle bits and Icelandic curb bits. On the other hand, horses ridden with a snaffle bit had 8 times higher odds for lesions in the buccal region (*P* = 0.0002) (Table 2) compared to all curb bits. No significant difference was found in the frequency of lesions between the different disciplines. The number of performances between the two examinations did not have a significant effect. All tests of two-way interactions were not significant.

Lesions were found on the lateral edges of the tongue in five horses, always in combination with severe findings on the bars of the mandible. All had been ridden with a curb bit which had a port in the previous performance.

## Discussion

The field study allowed examination of the most rostral part of the oral cavity, including the tongue and the bars of the mandible, which are the regions expected to be most prone to bit-related injuries [5]. The caudal regions of the oral cavity were not examined in this study. The horses were not re-examined directly after each performance, and thus some new (acute) lesions might have passed unnoticed. Re-examination of horses going further to the finals should, however, have detected all new lesions in this group of horses, except the most superficial ones that had already healed. In total, 8 veterinarians with special training performed the examinations. To ensure consistency, one veterinarian (SB), supervised all the examinations and the grading of severe findings was in most cases made in consensus between two veterinarians.

The frequency of oral lesions was already found to be high (43%) prior to the preliminary tests although most of the findings were graded as mild at that time (Table 2). Separate analyses were performed for the lesions in the two different locations (buccal and bars) as they were suspected to have different risk factors. The frequency of findings in the buccal region remained unchanged between the two examinations. A strong association between the buccal lesions and the use of snaffle bit was confirmed (Table 2) which is in agreement with earlier reports [5]. Lesions in the buccal region were also found in horses ridden with a curb bit which had a port indicating those horses may be trained in a snaffle bit.

The use of a curb bit with a port was found to be a decisive risk factor for lesions in the bar region. The increase in the frequency of lesions in this location between the first and second examination from 7.8% to 31.2% respectively (Table 3) demonstrated a competition effect, given that curb bits with a port had also been used (to some degree at least) in training both before and during the competition. Therefore, an even higher frequency could be expected after the finals. The high frequency of lesions in the bar region in horses competing in T1 (67%) was explained by the frequent use of a curb bit which had a port. The complete absence of lesions in horses competing in T2 is an interesting finding given that all were ridden on a curb bit with a port. It indicates the positive impact of both self-carriage and less critical head and neck position required for horses competing in the T2 discipline. This is supported with results from the first examination where horses competing in T2 and pace had a lower frequency of severe findings compared to the other disciplines. In pace competitions the emphasis is on speed rather than carriage and the horses are ridden without constant pressure from the reins, although severe rein aids are often seen temporarily in the transition from gallop to pace. This might explain the lower frequency of oral lesions in pace horses although the results are only based on one examination and not controlled for the type of bits used in that discipline.

Six horses ridden with traditional Icelandic curb bits were all without oral lesions which is also a remarkable finding. It suggests the port plays a major role in the development of lesions in the bar region. However, the leverage effect of shanks and curb is likely to increase the effect of the port.

The tongue has been described as a pad that acts to protect the bars of the mandible from the pressure of the bit and to control the distribution of bit pressure within the oral cavity [12]. On the other hand, it has been demonstrated how horses may frequently bulge the tongue over variations of the snaffle bit [13] which might increase the risk of lesions on the bars. That habit, however, should have been hindered in horses ridden in a bit with a port. The bar lesions were in most cases located caudal to the level of the *lingual frenulum* (Figure 2), which was unlikely to be possible with the tongue over the bit, and therefore the habit was excluded as a causative factor. It is postulated that the port of the bit circumvented the tongue’s protective capacity, by inhibiting it in lifting the bit, resulting in increased pressure directly to the bars. As the thin layer of mucosa and the sensitive periostium covering the mandibular bone cannot readily absorb the pressure while maintaining tissue integrity, the consequence would be painful tissue damage on the bars.

Lesions on the tongue were always seen together with severe findings in the bar region and were consequently thought to result from the horse’s effort to protect the bars of the mandible by squeezing the tongue between the bit and the bars as described by Johnson [7].

The English noseband which was always combined with the two types of curb bits (with and without a port) had no correlation with the development of lesions in the bar region. By contrast, the influence of the German noseband (or similar) on the occurrence of oral lesions cannot be separated from that of the snaffle bit.

Performance of the characteristic gait *tölt* is central to all disciplines developed for the Icelandic horse, except for the pace test and pace races. High front limb movements, together with an elevated head and neck position are among qualities that are rewarded [9]. Subsequently, a carriage close to the extremely elevated head and neck position described by Rhodin *et al.*[14] in the trot is frequently observed and contact to the mouth of the horse is allowed on a stretched rein. An extreme elevation of the head and neck has been shown in the trot to increase the (lumbar) back extension [14] which has been claimed to negatively affect the contact with the mouth of the horse [15].

Horses that qualified for the finals had commonly been ridden with a curb bit which had a port, especially in T1 (83%). This type of bit seems therefore to be of great importance in achieving the highest scores, most likely as the bit pressure on the bars helps the rider to achieve the rewarded head and neck carriage and to control the horse.

## Conclusion

It was concluded that bit-related lesions in the mouth are a general problem in Icelandic competition horses. The type of bit influenced both the location and the severity of the lesions. The use of curb bits with a port was found to be a decisive risk factor for lesions on the bars of the mandible, most of which were regarded as severe. The results also raised questions about the head and neck carriage demanded for the competition horses. From an animal welfare point of view, prevention of severe lesions in the bar region of the mouth should be given the highest priority.

## Competing interests

The authors declare that they have no competing interests.

## Authors’ contributions

SB designed the study, coordinated and performed clinical examinations, interpreted data and drafted the manuscript. RF conceived the study, helped to interpret data and draft the manuscript. TK interpreted data, performed statistical analysis and drafted the manuscript. TL provided professional advice, helped to interpret data and draft the manuscript. All authors approved the final manuscript.
